# Remission, complications, and overall survival in transsphenoidal pituitary surgery—a Swedish single-center experience of 578 patients

**DOI:** 10.1007/s00701-022-05456-8

**Published:** 2023-01-20

**Authors:** Ola Fridman Bengtsson, Ola Sunnergren, Ivan Segerhammar, Petter Förander, Martin Olsson, Anna-Lena Hulting, Pär Stjärne

**Affiliations:** 1 Department of Otorhinolaryngology, Karolinska University Hospital, Eugeniavägen 3, 171 76 Stockholm, Sweden; 2 Department of Clinical Sciences, Intervention and Technology, Division of Otorhinolaryngology, Karolinska Institute, Stockholm, Sweden; 3 Ear-, Nose-, and Throat Clinic, Jönköping County, Sweden; 4 Ear-, Nose-, and Throat Clinic, Örebro University Hospital, Örebro, Sweden; 5 Department of Neurosurgery, Karolinska University Hospital, Stockholm, Sweden; 6 Department of Molecular Medicine and Surgery, Patient Area Endocrinology and Nephrology, Inflammation and Infection Theme, Karolinska University Hospital, Karolinska Institute, Stockholm, Sweden

**Keywords:** Transsphenoidal, Pituitary surgery, Surgical results, Complications, Morbidity, Mortality, Survival

## Abstract

**Background:**

Surgical treatment of pituitary lesions causing hormonal overproduction or mass effect is standard procedure. There are few reports on the results and complications related to these surgeries from Northern Europe. Our aim was to evaluate the outcome and complications of a single tertiary surgical center over more than a decade.

**Methods:**

This was a retrospective study on all patients that underwent pituitary surgery from 1st of January 2005 to 31st of December 2017. The analysis included type of lesion, surgical method, pre- and postoperative need for hormonal substitution, hormonal outcome, complications to surgery, survival, need for revision surgery, or stereotactic radiation. Appropriate statistical analyses were made to evaluate surgical results, complications, and survival.

**Results:**

Five hundred seventy-eight patients were included in the study. Remission was achieved in 58% of patients with GH-producing and 94% of ACTH-releasing adenomas. Sixty-six percent had no preoperative hormonal substitution compared to 39% postoperatively. Rhinosinusitis (10%) was the most commonly reported postoperative complication followed by leakage of cerebrospinal fluid (8%) and meningitis (4%). Standardized mortality rate for the study population was higher (*p* = 0.18) when compared to the general population.

**Conclusion:**

Our results regarding remission rates and complications are in comparison with previous studies. Surgery of pituitary lesion can be considered a safe and efficient surgery. We noted lower rates of CSF leakage in the later part of the study period and believe that this, in part, was an effect by the introduction of a multidisciplinary surgical skull base team and increased surgical experience.

## Introduction

Pituitary adenomas (PA) are common, with a reported prevalence of 16.7% of intracranial tumors [9]. In addition to adenomas, other lesions originating from the pituitary may arise and provoke symptoms, most commonly cysts. PAs can be classified into hormone-releasing or hormonally inactive tumors (non-functioning pituitary adenomas, NFPA). Symptoms arise either due to affected hormonal production or compression of nearby structures, usually the optic chiasm [29]. PAs are also classified according to size, i.e., micro (< 10 mm), macro (> 10 mm), and giant adenomas (> 40 mm) [6, 16, 18]. With the exception for prolactinomas, surgery is the preferred treatment of PAs[29]. Pituitary surgery has undergone major changes over time, mainly due to technical development of surgical equipment. Nowadays, an endoscopic approach to the sellar region, with a dedicated skull base team, is commonly practiced[38]. The intricate anatomy surrounding the pituitary offers a surgical challenge and it is considered the main reason why non-radical resections are common [1]. A well-established complement to surgery, especially in Cushing disease, is stereotactic radio surgery (SRS)[5, 28, 32].

The literature contains numerous scientific publications on surgical results and techniques in endoscopic pituitary surgery[1, 4, 23, 29, 31]. However, few originate from northern Europe and none from Sweden, although these procedures have been performed here for many decades.

This study had multiple aims. Our first aim was to evaluate complication rates after primary transsphenoidal pituitary surgery in patients treated at Karolinska University Hospital 2005–2017. Secondarily, we wanted to evaluate survival rates in patients that have undergone transsphenoidal pituitary surgery of PA (both hormone-releasing PAs and NFPAs) compared to an age- and gender-matched general population. Thirdly, our aim was to evaluate remission rates (in hormone-releasing PAs), with or without complementary SRS and the need for postoperative hormonal substitution in patients with PAs.

## Materials and methods

### Study design

This was a retrospective observational study based on medical records from individual patients that underwent primary transsphenoidal pituitary surgery, with or without complementary SRS, due to assumed PA at the Karolinska University Hospital 1 Jan 2005 to 31 Dec 2017. The patients were identified by the surgical codes for transsphenoidal surgery of the pituitary gland and SRS.

Patients with craniopharyngeomas, germinomas, or chordomas were excluded as these are not considered true lesions of the pituitary gland. Patients who had either revision surgery at the Karolinska University Hospital or had postoperative follow-up at another hospital were also excluded from the study.

The medical records were reviewed for age, gender, date of surgery, date of death, hormonal release (pre- and postoperatively), hormonal substitution (pre- and postoperatively) type of surgery (± SRS), radiologic examinations, type of pituitary lesion (pathological-anatomical diagnosis), and postoperative complications.

Remission was defined as biochemical normalization of hormone hypersecretion (normal IGF, growth hormone (GH)) < 0.4 ng/mL after OGTT, and random GH < 1.0 ng/mL), normal Urinary Free Cortisol (UFC), and adequate inhibition after dexamethazone test. Detailed information regarding endocrinological follow-up is described in a later section.

Complications were defined as cerebrospinal fluid (CSF) leakage (verified using Beta transferrin protein test) requiring surgical treatment, postoperative sinonasal infection (signs of rhinosinusitis at follow-up endoscopic examination), bleeding which required intervention, diagnosed meningitis, sepsis, mortality or neurological deficits within 48 h from surgery, deep venous thrombosis (DVT), and pulmonary embolism (PE) during postoperative hospital stay.

All surgeries were performed in the Karolinska University Hospital, either at the Department of Neurosurgery or at the Department of Otorhinolaryngology, while the SRS was performed at the Department of Neurosurgery alone.

The date for the survival analysis was set to 18th of May 2018. Statistics for general population was collected from Statistics Sweden (www.scb.se).

The study was approved by the Central Ethical Review Board in Stockholm, Sweden (EBN 8,930,234).

### Surgical technique

All patients were operated in general anesthesia and received prophylactic perioperative corticosteroids (Solu-cortef ©) and a single dose of i.v. cephalosporine antibiotics. Patients allergic to beta-lactam antibiotics received i.v. clindamycine. The surgical method used was either a minimally invasive endoscopic transsphenoidal approach to the sella or transsphenoidal microscope-assisted surgery. In both methods, intracranial removal of adenoma was performed with cold-steel curettes and microforceps. In case of perioperative CSF leakage, this was sealed using autologous free fat graft (harvested from the lateral side of the right thigh) followed by one or more layers with absorbable hemostat (Surgicel©, Ethicon) and fibrin sealant (Tisseel©, Baxter).

### Follow-up

Postoperatively, all patients were followed up at the ENT department after 4–6 weeks for postoperative sinonasal endoscopic examination, evaluation of CSF leakage, and sinonasal status. Evaluation of the pituitary function was performed 4–8 weeks after the pituitary surgery. Prolactin, TSH (thyroid stimulating hormone), free T4 (thyroxine) and T3 (triiodothyronine), testosterone/estradiol, FSH (follicle stimulating hormone), LH (lutenizing hormone), and IGF-I (insulin-like growth factor) were analyzed. The HPA (hypothalamic–pituitary–adrenal) axis was evaluated with the short Synachten test. Function of the posterior pituitary lobe was assessed with measurement of urine osmolality. Remission of GH, ACTH, and Prolactin hypersecretion was assessed with OGTT (oral glucose tolerance test) and/or repeated measurements of GH in the morning and IGF-I, 24-h U-cortisol, repeated measurements during 24 h of S-cortisol, P-ACTH, saliva cortisol, dexamethazone test, and prolactin respectively. MRI was performed 3 months after surgery and discussed at multidisciplinary conference and patients were thereafter evaluated at the neurosurgery outpatient clinic.

### Statistical methods

Descriptive statistics and variate analyses were calculated using statistical software (SPSS, version 24, IBM©). Crosstabulation for odds ratios and chi^2^ test for bivariate analysis for complications were used and the Kaplan–Meier graphs and standardized mortality rate for comparison of survival rate, compared to general population where data was obtained and matched from Statistics Sweden.

## Results

In total, 578 patients that met inclusion criteria were identified. There were slightly fewer females 282 (49%) in the population. The mean age was 53±16 y. The general characteristics of the study population are presented in Table 1. In all patients, the indication for surgery was either a tumor mass effect on nearby structures, mainly optic chiasm, hormone-secreting adenoma, or a combination of the two. The majority of the pituitary adenomas were hormonally inactive 355 (61%), followed by GH-producing adenomas 104 (18%), and ACTH-producing adenomas 50 (9%).

**Table 1:** General characteristics of the study population

Study population, *n*	578
Mean age, mean (SD)	53±16
Gender, *n* (%)	
Female	282 (49)
Male	296 (51)
Lesion type, *n* (%)	
NFPA	355 (61)
GH	104 (18)
ACTH	50 (9)
PRL	20 (3)
TSH	4 (1)
Cyst	34 (6)
Other	11 (2)
Lesion size, *n* (%)	
> 10 mm	498 (86)
< 10 mm	80 (14%)
Surgical technique, *n* (%)	
Microscope	61 (11)
Endoscope	517 (89)
Stereotactic radio surgery	64 (11)

Up until early 2010, microscope-assisted surgery was performed in 61 patients; thereafter, the remaining 517 was treated purely endoscopically.

### Remission

The remission rates are presented in Table 2. In mono-hypersecretional PAs, remission was achieved with primary surgery in 49/104 (47%) of GH-producing PAs and 40/50 (80%) in ACTH-releasing PAs. Eleven patients with GH overproduction received complementary treatment due to either mass effect or persistent hypersecretion. Of these, 5 underwent a secondary surgery while 6 received SRS in order to achieve hormonal cure. In ACTH-releasing adenomas, 2 had complementary surgery and 5 received SRS until cured. Consequently, a total remission rate of 60 (58%) out of 104 patients and 47 (94%) out of 50 patients with GH- and ACTH-releasing adenomas respectively was achieved.

**Table 2:** Remission rates of hormone-releasing adenomas

Type	Primary surgery	Revision surgery	Surgery and stereotactic radio surgery	Total remission
GH	49/104 (47%)	5/104 (5%)	6/104 (6%)	60/104 (58%)
ACTH	40/50 (80%)	2/50 (4%)	5/50 (10%)	47/50 (94%)

### Hormonal substitution

Data on hormonal substitution is presented in detail in Tables 3 and 4. The majority of patients (380 (66%)) did not need hormonal substitution prior to surgery. However, the proportion of patients in need of substitution increased from 35% before treatment to 61% after surgery. The majority of patients (342 (59%)) had no alteration in the number of substituted hormones before and after treatment and 29 (5%) patients had a decreased need of hormone treatment after surgery.

**Table 3:** Pre- and postoperative hormonal substitution

Hormonal substitution	Preop, *n* (%)	Postop, *n* (%)
No axis	380 (66)	225 (39)
1 axis	111 (19)	167 (29)
2 axis	56 (10)	113 (20)
3 axis	28 (5)	62 (10)
4 axis	3 (1)	11 (2)

**Table 4:** Difference in hormonal substitution 6–8 weeks after transsphenoidal pituitary surgery

	*n* (%)
No difference in the number of substituted axis	342 (59)
One additional substituted axis	144 (25)
Two additional substituted axes	56 (10)
Three additional substituted axes	26 (4)
Four additional substituted axes	5 (1)
One less substituted axis	25 (4)
Two less substituted axes	4 (1)

### Complications

**Fig. 1:**
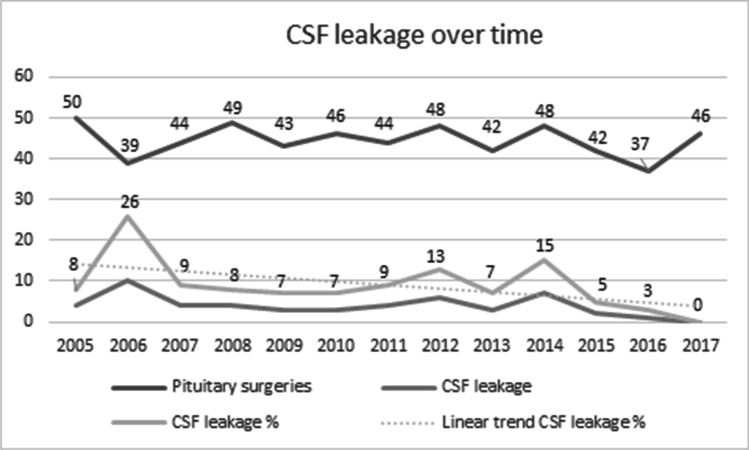
CSF leakage over time

The rates of complications are presented in Table 5. The most frequent complication was rhinosinusitis in 63 patients (10%). The second most frequent complication was CSF leakage in 51 patients (8.4%). The rates of CSF leakage decreased over the later period of the study period (Fig. 1).

**Table 5:** Surgical complications

Surgical complications	*n*	%
Rhinosinusitis	63	11
CSF leakage	49	8
Meningitis	26	4
Sepsis	12	2
Neurological deficits	5	< 1
Bleeding	4	< 1
DVT/PE	2	< 1
Surgical mortality < 48 h	2	< 1

Meningitis was recorded in 26 patients (4%), whereas postoperative sepsis was recorded in 12 (2%) of the cases. Neurological deficits related to surgery, postoperative bleeding, DVT/PE, and surgical mortality < 48 h were observed in 5 (< 1%), 4 (< 1%), 2 (< 1%), and 2 (< 1%) patients, respectively (Table 5).

There were no statistically significant differences for any of the studied complications after surgery regarding gender (female vs male), size of adenomas (micro vs macro), hormonal release (PAs vs NFPAs), or surgical method.

### Patient survival

**Fig. 2:**
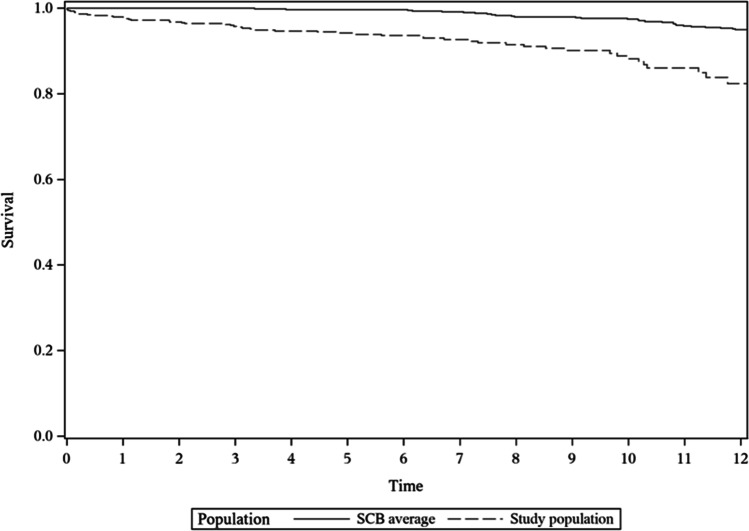
Kaplan–Meier graph showing survival over time as compared to general population (*n* 578)

The overall survival in the studied population is shown in Fig. 2. Comparison with the general population and study population was made through register data (SCB) and matched for age, gender, and place of residency. Standardized mortality rate for the study population over time was 1.2 (*p* = 0.18) as compared to the general population (Table 6) and 5- and 10-year survival was 94.2% and 88.2% respectively (Table 7).

**Table 6:** Standardized mortality rate (SMR) for the study population

Label	Number of observations	Observed person years	Observed events	Expected events	SMR value	95% CI	*p*-value
SMR	578	3777.4	53	43.5	1.21827	0.913–1.594	0.18

**Table 7:** 1-, 5-, and 10-year survival rates

Years	Survival rate	95& CI low	95% CI high
1	97.7%	96.1%	98.7%
5	94.2%	91.7%	95.9%
10	88.2%	83.9%	91.4%

## Discussion

This is, to our knowledge, the first Swedish study on overall remission and complications after surgery of pituitary adenomas. We found that our surgical results regarding remission in hormone-releasing adenomas were comparable to international published results[8] and that they improved by the combination with SRS. Our overall complication rates were generally low although CSF leakage was more common than expected. However, there was decreasing tendency in the later study period. Furthermore, we found that surgery of pituitary adenomas may increase the need for postoperative hormonal substitution. Finally, we found that the survival rate for the study population was slightly lower than that of the general population but comparable to other reported material from northern Europe[11]

In an international compilation of surgical results using endoscopic transsphenoidal approach from 2015, Dallapiazza et al. presented remission rates of 68–95% in ACTH-releasing adenomas. Using consensus remission criteria published in 2010[19], the surgical success rate varied from 46 to 70% in GH-producing adenomas overall[8]. Comparing our findings, we conclude that our results fall somewhere within the boundaries of previously published data regarding remission. In our study, primary surgery of ACTH- and GH-releasing adenomas accomplished remission in 80% and 47%, respectively. After revision surgery, remission was accomplished in 84% and 52%, respectively, and when combined with SRS, the cure rate was improved to 94% in ACTH-releasing adenomas and 56% in GH-releasing adenomas. SRS also showed to be an effective method in treating PA extending towards surgically challenging areas such as the cavernous sinus. According to the general opinion, SRS seems to be a viable option in the treatment of pituitary adenomas[5, 27, 34]. The risk of secondary brain tumors after SRS has been discussed in other studies, and although low, should be taken into consideration [20, 28, 30]. Too few other hormonally secreting tumors had undergone surgery to be conclusively assessed in our material [25].

Rhinosinusitis was the most commonly observed complication at first postoperative follow-up in our study. A fact for consideration is that 4 weeks after surgery, most patients have not achieved mucosal recovery. Additionally, our definition of RS was based either on patient complaints or objectives findings of crusting in the sinonasal cavity. Admittedly, this wide definition will probably over-estimate the number of patients with postoperative true bacterial infection. However, a number of publications have focused on PROMs including disease-specific quality of life instruments, relating to rhinosinusitis [2, 7, 13–15, 33, 36]. It seems that a major patient complaint, after pituitary surgery, is due to sinonasal morbidity, something that needs to be taken into account, when planning patients for surgery[2, 7, 33, 37, 40].

The rates of postoperative CSF leakage in our study seem to be high from an international perspective. However, the frequency of CSF leak varied over the years and in more recent years, our rates were comparable to those published from other high-volume centers [12, 24]. Several studies have indicated an advantage of using a multidisciplinary team consisting of rhinologists and neurosurgeons when performing endoscopic approach to the sellar region[23]. This method of surgical collaboration, probably in combination with increased surgical experience, has had a positive effect on surgical outcome and complication rate[35, 41]. Initially in our study period, surgery of PAs was performed in either the ENT or neurosurgery department separately, but in recent years, a dedicated “high volume,” multidisciplinary surgical skull base team has been formed that does nearly all the endoscopic surgeries together. This change of surgical cooperative paradigm seems to have had a positive effect on the surgical results.

The majority of patients in our study were treated with transsphenoidal endoscopic technique, which today is the most widely used and commonly accepted approach [25, 39]. Several studies have compared the surgical success between this technique and the previously more commonly used microscopical transsphenoidal approach and shown similar results[3, 15, 21, 22, 31].

An interesting finding in our study is the fact that a fairly large group of patients increased their need for postoperative hormonal substitution after surgery. The majority of these patients were treated with postoperative hydrocortisone as additional hormonal substitution and we know from a previous study that hydrocortisone treatment is more commonly used in our institution compared to international standards[10]. This fact may very well influence the true need for postoperative hormonal substitution, and should perhaps be analyzed in detail separately.

### Limitations

This was a retrospective investigation. However, the study population is large, and the time period long. Data regarding visual field was not achievable due to inconsistent/incomplete medical journals which was unfortunate, as this is a major indication for surgery on clinically inactive macroadenomas. In this study, we chose to classify the PAs based on clinically significant hormonal secretion. The major reason why, was that in our center, this classification was used to decide the treatment. Other reasons were the retrospective design and the long period (2005–2017) when data was collected. During this period, the WHO has proposed novel recommendations on the classification of PAs [17]. However, we acknowledge that a more detailed classification of PAs, such as the WHO or the classification proposed by Mindermann in 1997, could have strengthened the results [26]. Another limitation was the definition of rhinosinusitis where we, in our follow-up after pituitary surgery, did not use any standardized definition. Therefore, the quality of variables studied (including rhinosinusitis) is limited to what can be discerned from the medical records. This means that the relatively high rate of postoperative rhinosinusitis found in our study should be interpreted with caution as follow-up was made early after surgery, prior to complete mucosal recovery.

### Conclusion

In summary, this is the first Swedish study of overall surgical success and complications after surgery of pituitary lesions. When compared to international studies, our results regarding hormonal remission rates are comparable. The most commonly noted complication was rhinosinusitis, followed by CSF leakage. In the latter, we saw although a high overall rate, but with a positive trend towards lower rates in the later part of the study period. We believe that this, at least in part, was an effect of the introduction of a multidisciplinary surgical skull base team and increased surgical experience.

## Data Availability

The data that support the findings in this study is available from corresponding author, upon reasonable request.

## Abbreviations

PA: Pituitary adenoma
NFPA: Non-functioning pituitary adenoma
SRS: Sterotactic radiation surgery
OGTT: Oral glucose tolerance test
IGF: Insuline-like growth factor
GH: Growth hormone
ACTH: Adrenocorticotrophic hormone
CSF: Cerebrospinal fluid
UFC: Urinary free cortisol
DVT: Deep venous trombose
PE: Pulmonary embolism
ENT: Ear, nose, and throat
TSH: Thyroid-stimulating hormone
T4: Thyroxine
T3: Triiodothyronine
FSH: Follicle-stimulating hormone
LH: Lutenizing hormone
HPA: Hypothalamic-pituitary-adrenal
MRI: Magnetic resonance imaging
